# Long-lasting concordance between imaging-guided and clinical-based STN-DBS programming enhances motor and axial outcomes in Parkinson's disease: A 3-year single-center study

**DOI:** 10.1177/1877718X261428281

**Published:** 2026-07-07

**Authors:** Leonardo Rigon, Francesco Bove, Alessandro Izzo, Nicola Montano, Alessandro De Biase, Quintino Giorgio D’Alessandris, Danilo Genovese, Alice Calarco, Paolo Calabresi, Carla Piano, Anna Rita Bentivoglio

**Affiliations:** 1 Department of Neurorehabilitation, IRCCS San Camillo Hospital, Venice, Italy; 2 Padova Neuroscience Center (PNC), University of Padua, Padua, Italy; 3 Neurology Unit, Fondazione Policlinico Universitario A. Gemelli IRCCS, Rome, Italy; 4 Department of Neuroscience, Università Cattolica del Sacro Cuore, Rome, Italy; 5 Neurosurgery Unit, Fondazione Policlinico Universitario A. Gemelli IRCCS, Rome, Italy

**Keywords:** deep brain stimulation, image-guided programming, axial symptoms, speech disturbances, directional stimulation, subthalamic nucleus

## Abstract

**Introduction:**

Subthalamic deep brain stimulation (STN-DBS) improves Parkinson's disease motor symptoms. Directional leads can extend the therapeutic window, despite further increasing programming complexity. Lead placement visualization software can facilitate STN-DBS programming, especially in contact selection process. We investigated the extent and impact on motor outcomes of the concordance between imaging-suggested (IGP) and standard clinical-programming (CP) selected stimulation contacts three years after surgery.

**Methods:**

Thirty-five PD patients with bilateral STN-DBS were enrolled. Lead localization was reconstructed using Brainlab™. For each electrode, the predicted optimal vertical contact and, when applicable, directionality was identified and compared with stimulation parameters clinically activated three years post-surgery. Concordance, agreement and similarity IGP/CP metrics were calculated for both contact level and directionality. Postoperative changes in motor symptom severity were compared between concordant and discordant IGP/CP groups.

**Results:**

Three years after surgery, IGP/CP concordance was 77.6% for active stimulation contact level and 66.7% for directionality. IGP reliably identified contacts and directional segments avoided by CP for chronic stimulation (negative predictive value 0.85 and 0.72, respectively). No significant difference was found in motor outcomes based on IGP/CP contact level concordance. Nonetheless, superior benefit in overall motor function and axial impairment (including speech) were reported in patients with clinically-activated directional stimulation concordant with that suggested by visual reconstruction.

**Conclusions:**

Visualization software can simplify STN-DBS programming, especially by reducing the number of contacts warranting systematic clinical testing via monopolar review. Moreover, the combination of visualization and directional stimulation technologies can further improve outcomes, especially of those symptoms, like axial/speech impairment, often challenging subthalamic chronic stimulation.

## Article highlights:

- We found a considerable concordance rate (77.6%) between imaging-suggested optimal stimulation contacts and those activated three years after surgery following standard monopolar review and clinical programming;
- Imaging reliably identified contacts and directional segments that were not chosen by clinical programming for chronic stimulation;
- Patients with activated directional segments concordant with those suggested by visual reconstruction showed increased stimulation-induced benefit in overall motor function and axial symptoms (including speech).

## Introduction

Deep brain stimulation (DBS) of the subthalamic nucleus (STN) is an established therapy in Parkinson's disease (PD).^1,2^ Overall, STN-DSB provides substantial benefit on PD motor symptoms^3^; however, certain disturbances (e.g., axial and speech impairment) often exhibit only limited or transient response and may even worsen under stimulation, despite optimized DBS programming.^4,5^ Currently, clinical programming (CP) represents the gold standard for selecting DBS parameters. The initial activation is guided by a monopolar review (MoR), which consists of the systematic clinical evaluation of each contact for both electrodes.^6^ However, this procedure is time-consuming and often burdensome for patients, as repetitive testing can reduce compliance and fatigue may hinder reliable assessment.

The selective stimulation of the dorsolateral portion of the STN has been associated to improved motor outcomes.^7,8^ In this light, directional leads (shaped with the two middle ring contacts subdivided into three individual segments each) allow to steer the current toward the optimal target while minimizing spread to adjacent structures,^9^ thereby increasing efficacy and reducing side effects.^10–12^ Yet, these technological advances further increased programming complexity, given the exponential growth in possible contact configurations to test during MoR. In this context, imaging-guided programming (IGP), which visualizes lead placement in relation to patient-specific anatomy, can facilitate contact selection by identifying those oriented toward the dorsolateral STN. This approach has been linked to motor outcomes comparable to CP, despite significantly reduced programming time.^13–15^ Moreover, visualization of lead placement is particularly valuable when reprogramming patients with suboptimal postoperative improvement and/or stimulation-induced side effects following CP.^14,16^ The concordance rate between contacts suggested by IGP and those selected by CP for chronic stimulation (6–12 months) has been reported to be around 60–80%^17–20^; moreover, IGP/CP agreement has been recently associated to superior motor outcomes when using directional stimulation.^21^ However, the extent and clinical relevance of IGP/CP concordance in longer follow-up periods remains to be clarified. This aspect is particularly relevant given the challenges that prolonged CP faces due to delayed-onset stimulation-induced side effects and/or stimulation-resistant symptoms. In this light, we aimed to investigate the concordance between the CP-selected and IGP-suggested optimal contacts in PD patients three years after STN-DBS, and evaluate its relation to motor outcomes.

## Materials and methods

This retrospective observational study adhered to the methodology described in our previously published work to ensure methodological consistency.^21^

In this study, PD patients treated with STN-DBS and available 3-year follow-up evaluation were enrolled. Inclusion criteria were diagnosis of PD according to the United Kingdom Parkinson's Disease Brain Bank criteria,^22^ eligibility to surgical interventional therapies for PD according to CAPSIT criteria,^23^ bilateral STN-DBS implant, active follow-up three years after surgery, and availability of lead placement reconstruction. Exclusion criteria were suboptimal pre-operative MRI/post-operative CT quality interfering with 3D reconstruction protocol and unavailability of stimulation parameters at 3-year follow-up.

Detailed information regarding the surgical procedure can be found in Supplementary Methods. Briefly, all electrodes were placed using the NexFrame system (Medtronic, Inc., Minneapolis, MN)^24^ in awake surgery. Leads placement was monitored intraoperatively with microelectrode recordings and imaging acquisitions; furthermore, microstimulations were delivered to explore the therapeutic window.

Electrode placement was visually reconstructed using Brainlab^TM^ (Brainlab AG, Munich, Germany). We decided to use Brainlab^TM^ for this study since it is formally approved for clinical use, as opposed to other popular freely available visualization software. Brainlab^TM^ localizes the leads in the patient-specific anatomy by co-registering preoperative MRI (1.5 T; 1mm-sliced whole brain, T1-weighted sequences + T2-weighted sequences covering from the top of thalamus to base of substantia nigra + SWI sequences) with postoperative CT scan. In particular, a thin-slice CT was obtained > 3 months after surgery to minimize pneumocephalus/edema brain shift artifacts following Brainlab^TM^-specific instructions (acquisition slice thickness ≤1 mm with no gap between slices; reconstruction slice thickness ≤ 0.625 mm; soft tissue reconstruction kernel). The acquisition of a delayed postoperative CT scan is in general clinically indicated to document the complete resolution of post-surgical structural alterations; in particular, the thin-slice protocol (as compared to standard acquisitions) did not significantly prolong the procedure time, and the adjunctive irradiation was negligible yet justified (∼ 0.5 mSv, comparable to approximately two months of natural background radiation exposure). Lead localization using Brainlab^TM^ software consists of three consecutive steps (total duration of the process: ∼ 30–40 min):

1. Image Fusion (Brainlab AG, Munich, Germany): automatically coregisters patient's preoperative MRI and postoperative CT;
2. Elements Anatomical Mapping (Brainlab AG, Munich, Germany): visualizes patient specific anatomy and runs an MRI-based subcortical segmentation;
3. Elements Lead Localization (Brainlab AG, Munich, Germany): detects the leads’ localization and, when applicable, orientation based on postoperative CT artifact and provides 3D visualization of lead contacts within the patient's own anatomy of target structures.

All steps were visually inspected by our team and minor corrections to the automated process output were performed when needed. For directional systems, orientation of the marker was defined by Brainlab^TM^ according to CT artifact and was confirmed with two orthogonal projections skull X-ray.

Initial activation following the MoR, as well as subsequent chronic programming, was performed by movement disorder specialists from our centre blinded to the 3D-visualization of electrodes placement ∼3 weeks postoperatively with the patient in OFF-medication state: the procedure lasted on average approximately 50–70 min. Chronic CP sessions were repeatedly performed throughout the 3 years follow-up (∼ 11–14 total visits). Their schedule was determined on a case-by-case basis according to individual clinical evolution. Generally, per standard practice at our centre CP visits are initially performed monthly then gradually spaced to every 4 months: their average duration is highly dependent on the complexity of the individual case but can be estimated around 20–40 min per session. The study was approved by the local Ethics Committee and all enrolled patients provided their informed consent.

### IGP/CP concordance analysis

For the purpose of IGP/CP concordance assessment, ring contact levels located within the STN and directional segments oriented toward its dorsolateral subregion were defined as IGP-suggested, and subsequently compared with those selected by CP and chronically activated at the 3-year follow-up. If two contacts were localized inside the STN, they were both considered IGP-optimal, as in a previous literature.^19,21^ For patients with bipolar or double monopolar configurations activated at follow-up, we considered the electrode to be IGP/CP concordant when >50% of the activated tissue, as simulated by the Brainlab^TM^ pipeline, resided within the STN.^21^ Based on these principles, two independent raters (L.R. and F.B.) visually inspected all electrodes and categorized them into IGP/CP-concordant and -discordant; in case of disagreement, a senior Author was consulted (C.P.).

Patients were classified according to contact level concordance into IGP/CP-concordant (c-Contact) and IGP/CP-discordant (d-Contact). Likewise, among patients receiving active directional stimulation, two groups were identified: concordant (c-Direction) and discordant (d-Direction). In case of suboptimal concordance (i.e., IGP/CP agreement on only one of the two electrodes) the patient was categorized based on the concordance status of the lead contralateral to the more severely affected body side.^21^ Notably, misplaced leads (i.e., outside the STN) were excluded from the analysis. Similarly, patients presenting any lead localized outside the STN were excluded.

### Clinical assessments

The following data were collected at baseline and three-year after surgery: levodopa equivalent daily dose (LEDD),^25^ Hoehn and Yahr (H&Y)^26^ score and full Unified Parkinson's Disease Rating Scale (UPDRS).^27^ Motor function (UPDRS-III total score) was sequentially evaluated under all possible therapeutic conditions (ON/OFF-medication at baseline; ON/OFF-medication/stimulation at follow-up).^21^ For further analysis, the UPDRS-III axial subscore, defined as the sum of items 18 (speech), 22 (neck rigidity), 27 (arising from chair), 28 (posture), 29 (gait), and 30 (postural stability) was extrapolated^28^: in this context, a common pathophysiological basis underlying speech, gait and postural disturbances in PD has been repeatedly reported.^29^

### Statistical analysis

Concordance rates for contact level and directionality were calculated as percentages (*(100×IGP/CP-concordant) / total*). Furthermore, each contact level and, when applicable, each directional segment, was individually assessed and dichotomized as CP-selected (yes/no) and IGP-suggested (yes/no). From these data IGP accuracy, sensibility, specificity, positive predictive value (PPV), negative predictive value (NPV), Cohen's k and Dice coefficient were calculated.

Baseline clinical and demographic characteristics were compared using the Mann-Whitney test while categorical variables were analysed with the chi-square test.

Changes in clinical scales scores between baseline and follow-up were compared with the paired-samples Wilcoxon-signed rank test. To compare DBS outcomes between concordant vs discordant patients, a linear regression model was fitted with clinical variation between two time points (*Δ variable* *=* *follow up value - baseline value*) as the dependent variable, group as predictor, and sex and age as covariates (enter method). All statistical computations were two-tailed, and a p-value < 0.05 was considered significant. The statistical analyses were performed using JASP (*version 0.18.3, jasp-stats.org*).

## Results

### Study population and DBS efficacy

Thirty-five patients (24 men), accounting for 70 total leads, were included. Ten patients had a Medtronic 3389 non-directional system, whereas the remaining 25 subjects had a directional DBS implant (3 Abbott directional and 22 Boston Scientific Cartesia). One serious adverse event occurred (lead replacement due to infection). Clinical and demographic characteristics of our study population at baseline and three years after surgery are summarized in Table 1. Significant (*p* *<* *0.001*) stimulation-induced improvement of motor symptoms severity (UPDRS-III and IV scores), as well as LEDD reduction, were reported at the 3-year follow-up as compared to baseline (Table 1). However, stimulation-induced improvement in the axial subscore (*baseline OFF-medication vs follow-up OFF-medication/ON-stimulation)* did not reach statistical significance (*p* *=* *0.06*); additionally, axial symptoms significantly worsened at follow-up as compared to baseline in both best-ON (*baseline ON-medication vs follow-up ON-medication/ON-stimulation*) and worst-OFF (*baseline OFF-medication vs follow-up OFF-medication/OFF-stimulation*) conditions.

**Table 1.** Demographic and clinical features at baseline and three-year follow-up of the study population. All comparisons were carried out using the Wilcoxon-signed rank test. * stimulation-induced changes were calculated comparing the ON-stimulation/OFF-medication state with the OFF-medication baseline condition. Abbreviations: UPDRS = Unified Parkinson's Disease Rating Scale; LEDD = Levodopa Equivalent Daily Dose.

Variable, mean (± SD)	Baseline	One-year follow-up	*p*
Age at DBS implant	56.3 (± 7.4)		
Disease duration at DBS implant	11.2 (± 5.3)		
Male sex, n/N (%)	25/35 (71.4%)		
Hoehn & Yahr	2.8 (± 0.6)	2.6 (± 0.5)	0.2
UPDRS I total	1.5 (± 1.2)	2.6 (± 2.2)	**0**.**005**
UPDRS II total	12.2 (± 5.5)	12.8 (± 6.1)	0.4
UPDRS III			
Best-ON	18.7 (± 7.2)	18.1 (± 10.5)	0.3
Worst-OFF	39.1 (± 14.1)	43.8 (± 11.8)	**0**.**009**
ON-stim/OFF-med		25.8 (± 10.3)	**<0**.001*
UPDRS III axial subscore			
Best-ON	4.2 (±2.3)	5.1 (±2.7)	**0**.**029**
Worst-OFF	8.8 (± 4.9)	10.3 (±3.4)	**0**.**017**
ON-stim/OFF-med		7.0 (± 2.9)	0.069*
UPDRS IV total	7.03 (± 3.41)	3.21 (± 1.94)	**<0**.**001**
LEDD	1302.59 (± 482.93)	832.51 (± 356.33)	**<0**.**001**

### Stimulation parameters and lead placement

Three years after surgery, directional stimulation was activated upon clinical judgement in 17 patients (22 electrodes). DBS configurations active at follow-up can be found in Supplementary Table 1.

**Figure 1.**
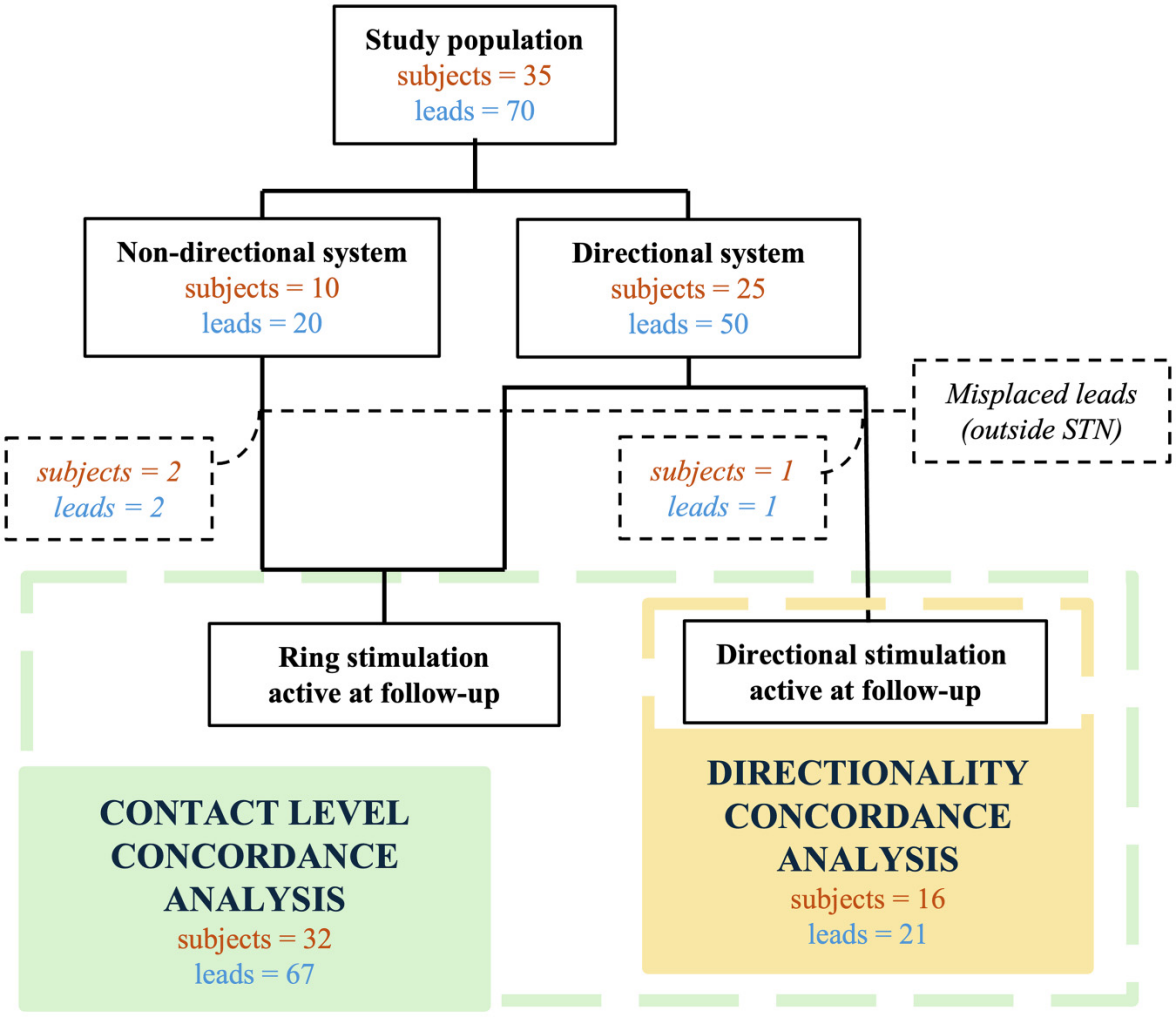
Flowchart of the study. After the exclusion of three leads (belonging to three patients), the contact level concordance analysis was performed on the whole study population, while the directionality concordance analysis was only performed on patients (and leads) with an active directional stimulation configuration at the three-year follow-up visit.

Automated 3D-visual reconstruction of the lead placement was successfully carried out for all subjects. Three misplaced leads were documented, one of which was activated with a directional configuration at follow-up: these electrodes (and the three respective patients) were therefore excluded from the analyses (a detailed flowchart of the study population is shown in Figure 1). Specifically, among these three patients: the first subject responded poorly to DBS (*Δ* LEDD = 625; *Δ* UPDRS-III OFF-medication = −3); the second reported benefit on motor symptoms although requiring increased oral dopaminergic treatment (*Δ* LEDD = 527.5; *Δ* UPDRS-III OFF-medication = −16); finally, in the third case surgical outcomes were overall satisfactory (*Δ* LEDD = 0; *Δ* UPDRS-III OFF-medication = −14).

### Concordance analysis

**Figure 2.**
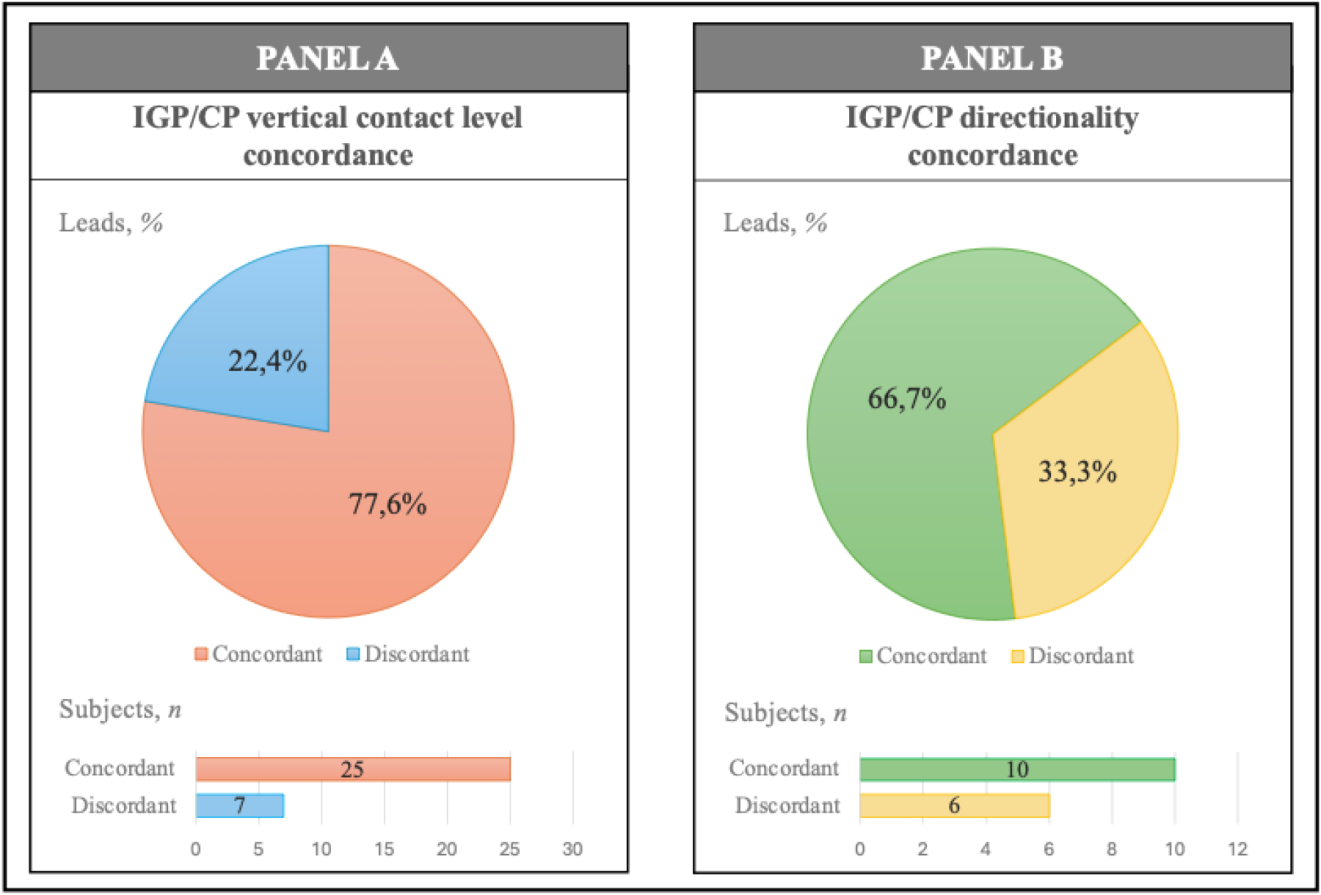
IGP/CP concordance analysis for vertical contact level (A) and directionality (B). In each panel, IGP/CP concordance regarding leads is displayed on top while that regarding subjects is depicted on the bottom. CP: clinical programming; IGP imaging-guided programming.

In our cohort, 52/67 leads (77.6%) and 25/32 subjects (78.1%) presented IGP/CP concordance in the contact level selection (Figure 2A), while IGP/CP concordant directionality selection was observed in 14/21 electrodes (66.7%) accounting for 10/16 patients (62.5%) (Figure 2B).

Moderate *(k* *=* *0.41)* and fair *(k* *=* *0.22)* IGP/CP agreement rates were reported for contact level and directionality selection respectively. The NPV was 0.85 for contact level and 0.72 for directionality prediction. Additional IGP/CP similarity indexes, as well as IGP performance metrics, are reported in Table 2.

**Table 2.** Imaging-guided programming performance metrics and agreement with clinical programming (current gold standard for stimulation contact selection). Abbreviations: PPV: positive predictive value; NPV: negative predictive value.

Metric	Contact level concordance (N = 268)	Directionality concordance (N = 63)
Accuracy	0.71	0.62
Sensibility	0.75	0.61
Specificity	0.69	0.62
PPV	0.54	0.50
NPV	0.85	0.72
Cohen's k	0.41	0.22
Dice coefficient	0.63	0.55

### DBS outcomes comparison according to contact level concordance

The total UPDRS-III scores significantly differed (*p* *=* *0.042*) at baseline between the two groups (c-Contact vs d-Contact); thus, this was included as covariate in the regression model. No difference was found in the remaining clinical and demographic variables (Supplementary Table 2).

Our regression model was not significant when analyzing the stimulation-induced changes in outcome variables, including motor improvement and LEDD reduction (Supplementary Table 3).

### DBS outcomes comparison according to directionality concordance

Among the two groups (c-Direction vs d-Direction) a significant gender imbalance was observed (*p* *=* *0.037*): this was accounted for in our multivariate model introducing gender as covariate. There were no other significant differences at baseline between groups (Supplementary Table 4).

**Figure 3.**
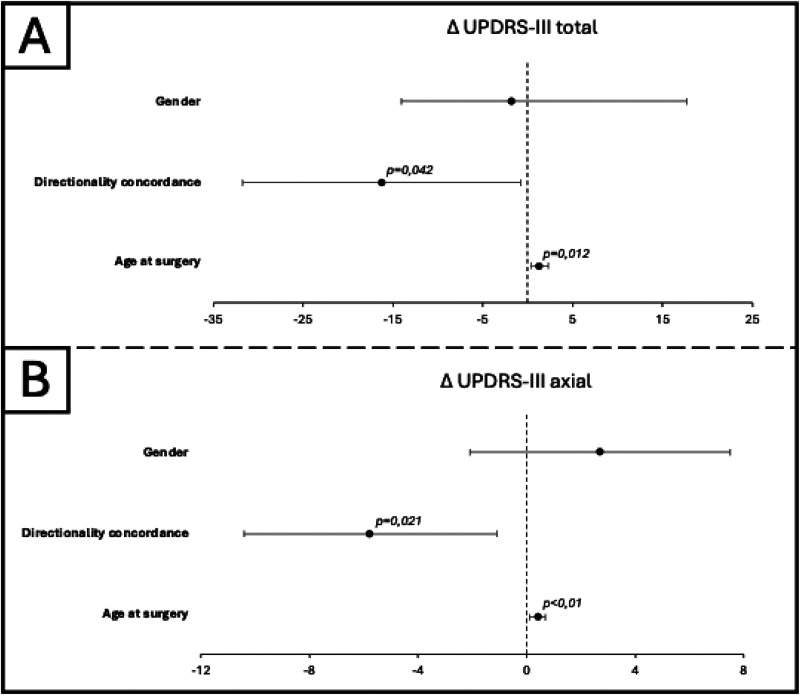
Impact of gender, directionality concordance and age at surgery on DBS motor outcomes in our cohort. Changes in motor assessments are expressed as Δ-variable = follow-up – baseline (negative values express greater postoperative reductions). Directionality concordance enhanced stimulation-induced control of both motor function (Panel A: Δ UPDRS-III) and axial symptoms (Panel B: Δ UPDRS-axial), whereas older age exhibited a detrimental effect; gender had no significant effect on the model.

Our regression model was significant when analyzing changes between baseline and follow-up scores of the total (*F(3,10)* *=* *4.2; p* *=* *0.036; adjusted-R^2^* *=* *0.4*) and axial (*F(3,10)* *=* *4.4; p* *=* *0.033; adjusted-R^2^* *=* *0.4*) UPDRS-III scores (Supplementary Table 3); in both cases, directionality concordance independently predicted greater stimulation-induced improvement (*UPDRS-III total: B* *=* *-16.2; SE* *=* *6.9; t* *=* *-2.3; p* *=* *0.042. UPDRS-III axial: B* *=* *-5.8; SE* *=* *2.1; t* *=* *-2.7; p* *=* *0.021*). Notably, age at surgery was also associated with changes in such measures, albeit in a negative direction (Figure 3).

## Discussion

To our knowledge, this is the first study investigating the IGP/CP concordance three years after surgery. While previous analyses focused on the short-term follow-up (≤12 months),^17–21^ our extended observational period allowed us to better account for the sustained effects of DBS. In clinical practice, neurologists often face delayed-onset side effects and stimulation-resistant symptoms,^3,4^ which remain difficult to adequately manage despite repeated programming sessions. Among these, speech and axial disturbances, alongside cognitive impairment, are particularly burdensome, given their substantial impact on patients’ functional status and quality of life.^30,31^ Notably, the effects of DBS on these domains have been consistently reported to decline over time^4^; moreover, a potential direct detrimental impact of stimulation on these features has been hypothesized.^5,32^ In this context, we aimed to assess the degree of IGP/CP concordance three years after surgery, with the goal of further clarifying its potential to facilitate DBS programming and its impact on motor symptoms, especially on those that become increasingly challenging under prolonged subthalamic stimulation.

In our cohort we observed a substantial IGP/CP concordance rate (77.6%) and moderate/good agreement (Cohen's k = 0.42) and similarity (Dice coefficient = 0.63) regarding contact level selection: these findings are consistent with previously published works on shorter follow-up periods (6–12 months).^17,18,20,21,33,34^ Taken together, these findings indicate that IGP can reliably identify the optimal stimulation contact level determined through CP even over extended follow-up periods. In clinical practice, this translates into greater time efficiency for neurologists and improved comfort for patients, owing to the reduced number of clinical evaluations and MoR required for chronic DBS programming. As a matter of fact, in our practice the standard MoR took almost twice as long as the full lead visualization procedure, in line with reports from other centres^13^; additionally, a considerable amount of time could have been saved during chronic refinement follow-up visits thanks to the availability of anatomical placement information. Furthermore, IGP minimizes the influence of motor fluctuations, fatigue, and raters’ subjective bias on programming sessions.

Fair IGP/CP agreement on directional segments selection was observed (concordance rate=66.7%; Cohen's k = 0.21; Dice coefficient = 0.55), albeit lower than that observed for contact level choice. This result is consistent with findings reported one year after DBS implant.^21^ Interestingly, the concordance rate at three-year follow-up was higher than one-year after surgery (66.7% vs. 51.7%),^21^ suggesting that, with repeated assessments, CP may progressively converge toward the IGP-suggested directional segment.

Several factors could contribute to the limited IGP/CP directionality concordance observed in our cohort. First, MoR systematically assessing all directional segments require particularly prolonged programming sessions, which are often poorly tolerated by patients in the OFF-medication state. This increases the impact of fatigue and discomfort on the evaluation: for these reasons we do not routinely perform a systematic MoR of directional segments upon initial activation, as commonly seen in clinical practice. Moreover, the limited time usually available for routine visits in clinical practice increases the risk of incomplete washout of stimulation effects from the previously tested segments. These issues could be particularly relevant in electrodes accurately positioned within the STN, where directional contacts often provide comparable motor efficacy, thereby amplifying the influence of the aforementioned biases.

The IGP PPV observed in our cohort was relatively low (0.54 and 0.50 for contact level and directionality prediction respectively). In our cohort, 55/70 electrodes presented two contacts within target; similarly, in some leads with active directional stimulation (9/21) two segments showed comparable proximity to the dorsolateral STN based on visual reconstruction. Accurate surgical lead placement often results in multiple contacts positioned within the target, due to their short length (1.5 mm each, separated by 0.5 mm spacing) relative to the dorsoventral diameter of the STN (∼5 mm)^35^: as stated in the Methods section, in these cases both contacts/segments were considered IGP-suggested. Considering two well-localized contacts as IGP-suggested is based on sound clinical reasoning: in fact, in everyday practice physicians would rarely avoid testing both contacts in such cases. Furthermore, this approach has been repeatedly employed in literature.^19,21,36^ However, since DBS programming was typically performed preferring single-monopolar or single-directional configurations, as is common in clinical practice, this approach resulted in a high false positive rate. Conversely, IGP showed good NPV for both contact level and directionality prediction (0.85 and 0.72 respectively), indicating that it can reliably identify contacts and segments that are avoided by CP during chronic subthalamic stimulation. Focusing on the true negatives, in our cohort IGP would have (correctly) discouraged the formal assessment of 124 (out of 268 total) contacts and 24 (out of 63 total) directional segments, thus significantly streamlining programming sessions by nearly halving the number of clinical evaluations required. Taken together, these findings offer important insights into the potential applications of IGP in clinical practice. This approach reveals to be particularly useful for identifying contacts/segments that are less likely to be effective on motor symptoms (i.e., those farther from the sensorimotor STN) in the context of chronic stimulation. Such information could guide MoR and subsequent chronic refinements, allowing clinicians to prioritize the systematic assessment of the accurately placed contacts/segments, especially given the aforementioned issues challenging the feasibility and reliability of repeated, prolonged programming sessions. In this light, the relatively low PPV should not be considered as a major limitation of IGP, as clinicians would rarely omit objective assessment of a contact located within the STN in routine practice. The same applies in cases with two well-positioned contacts/segments, where clinical evaluation remains essential to determine which one is the most beneficial.

In our cohort we did not find significant difference in motor outcomes between patients with and without contact level IGP/CP concordance, confirming the previously published results from the one-year follow-up analysis.^21^ This finding suggests that IGP may offer comparable efficacy to CP while substantially reducing programming time, particularly in the context of ring stimulation configurations. Conversely, IGP/CP directionality concordance was associated with greater stimulation-induced motor improvement (*Δ UPDRS-III*), again confirming results from the one-year follow-up.^21^ Furthermore, at the three-year follow-up we observed an additional improvement in axial disturbances among patients with IGP/CP-concordant chronic directional stimulation settings. Several definitions of axial subscore of the UPDRS III scale have been proposed in literature.^4,37–39^ In our analysis we considered the combination of items assessing speech, neck rigidity, gait, posture, postural stability and arising from chair, as these symptoms are typically less responsive to / tend to further decline under chronic subthalamic stimulation; furthermore, this particular subscore was used in prior works on bilateral STN-DBS in PD.^28^ In this context, our findings may reflect enhanced DBS efficacy, reduced stimulation-induced side effects or, more likely, a combination of both, resulting from more accurate stimulation of the sensorimotor subregion of the STN. Explaining the specific contribution of these mechanisms to the improvement of axial symptoms in IGP/CP concordant directional configurations goes beyond the scope of this work: further studies on larger cohorts may help to clarify this topic. Overall, we speculate that visualization technologies, particularly when applied to directional stimulation, may be especially valuable in managing symptoms that are typically difficult to control with chronic DBS, such as speech and axial impairment. This becomes even more relevant at prolonged follow-up, as these disturbances tend to progressively worsen over time despite optimized stimulation.^3,4,40,41^ Accordingly, in our cohort we observed no significant stimulation-induced improvement of the UPDRS-III axial subscore at follow-up as compared to baseline; moreover, axial impairment in the “best-ON” condition (*baseline ONmedication vs follow-up ONmedication/ONstimulation*) was significantly worse three years after surgery. Taken together, these findings support the hypothesis that speech and axial disturbances respond only partially to DBS and, over time, their deterioration due to disease progression (or possibly the detrimental effects of chronic subthalamic stimulation) is not adequately controlled despite optimized programming. Finally, as expected, older age impacted negatively the stimulation-induced improvement in both overall motor function and axial disturbances^42^; on the other hand, gender did not affect significantly motor outcomes in our model.

Overall, in our cohort we observed surgical safety and efficacy outcomes typical of DBS populations.^2,43,44^ These included significant stimulation-induced improvements in motor function and fluctuations, as well as LEDD postoperative reduction, despite the underlying disease progression reflected by the significant worsening in the “worst-OFF” condition (*baseline OFF-medication vs follow-up OFF-medication/OFF-stimulation*). These findings support the accuracy of patient selection, targeting, and programming processes in our study.

The main limitation of our study is the limited sample size: thus, further studies on larger populations are warranted to confirm our findings. Furthermore, owing to the retrospective design of the study, we lacked an IGP-based refinement in IGP/CP discordant cases. Finally, only ring configurations were systematically tested in the MoR prior to initial activation, while individual directional segments were evaluated in the follow-up just in patients requiring directional stimulation due to clinical indications (e.g., suboptimal efficacy, stimulation-induced side effects). This approach, although possibly more representative of real-world outpatient DBS programming, could underestimate the additional improvements that may be achievable with directional stimulation in some patients already benefitting from ring configurations.

The major strength of our study is the prolonged observational period (three years). Previously published literature in this field is limited and considered shorter (6–12 months) follow-up timepoints.^17–21^ Our study expands current knowledge on the role of lead placement visual reconstruction in chronic subthalamic stimulation, underscoring its clinical relevance particularly in relation to motor symptoms that often challenge long-term DBS programming (e.g., speech and axial disturbances). Moreover, we used for this study thin-sliced CT scans obtained > 3 months after surgery, thus overcoming the previously reported limitation of pneumocephalus and brainshift artifacts which can affect lead placement reconstruction accuracy.^19^

In conclusion, our findings support the implementation of lead placement visual reconstruction software in clinical practice, highlighting its valuable role in guiding MoR and subsequent refinements by reducing the number of contacts requiring systematic evaluation by the clinician. Moreover, our results suggest that imaging-informed directional stimulation may further broaden the therapeutic window in chronic DBS, particularly when addressing symptoms that typically remain challenging in the long term, such as speech and axial disturbances.

## Supplemental Material

sj-docx-1-pkn-10.1177_1877718X261428281 - Supplemental material for Long-lasting concordance between imaging-guided and clinical-based STN-DBS programming enhances motor and axial outcomes in Parkinson's disease: A 3-year single-center study Supplemental material, sj-docx-1-pkn-10.1177_1877718X261428281 for Long-lasting concordance between imaging-guided and clinical-based STN-DBS programming enhances motor and axial outcomes in Parkinson's disease: A 3-year single-center study by Leonardo Rigon, Francesco Bove, Alessandro Izzo, Nicola Montano, Alessandro De Biase, Quintino Giorgio D'Alessandris, Danilo Genovese, Alice Calarco, Paolo Calabresi, Carla Piano and Anna Rita Bentivoglio in Journal of Parkinson's Disease
