# Supplementary material for: Long-lasting concordance between imaging-guided and clinical-based STN-DBS programming enhances motor and axial outcomes in Parkinson's disease: A 3-year single-center study
Source: J Parkinsons Dis. 2026 Jul 7:1877718X261428281. Online ahead of print. doi: 10.1177/1877718X261428281 (PMC13435167; doi:10.1177/1877718X261428281)
Supplement: Supplementary file 1 [file sj-docx-1-pkn-10.1177_1877718X261428281.docx]

Supplementary Table 1: Active stimulation parameters at the three year follow-up. Misplaced leads (thus excluded from the analyses) are highlighted in bold. Contact levels are numbered craniocaudally 1 (most dorsal) to 4 (most ventral); directional segments are listed as A (2/5), B (3/6) and C (4/7). Imaging-based suggested directionality was only explored in leads with active directional stimulation in line with the concept of the concordance study. Abbreviations: Sbj = subject; Amp = amplitude; PW = pulse width; Fq = Frequency; N/A = not applicable.

| **Sbj** | **Lead type** | **Side** | **Configuration** | **Active contacts** | **Amp (mA)** | **PW (µs)** | **Fq (Hz)** | **Imaging-suggested contact level** | **Imaging-suggested directionality** |
| --- | --- | --- | --- | --- | --- | --- | --- | --- | --- |
| 1 | Medtronic 3389 | Left | Double monopolar | 0neg 1neg | 2.9 | 60 | 150 | 2-3 | N/A |
| 2 | Medtronic 3389 | Left | Single monopolar | 1neg | 2 | 60 | 150 | 1 | N/A |
| 3 | Medtronic 3389 | Left | Bipolar | 1neg 2pos | 2.8 | 60 | 180 | 3-4 | N/A |
| 4 | Medtronic 3389 | Left | Double monopolar | 1neg 2neg | 3 | 60 | 180 | 3-4 | N/A |
| 5 | Medtronic 3389 | Left | Single monopolar | 1neg | 2.8 | 60 | 130 | 3-4 | N/A |
| 6 | Medtronic 3389 | Left | Double monopolar | 2neg 3 neg | 4.6 | 60 | 60 | 3 | N/A |
| 7 | Medtronic 3389 | Left | Double monopolar | 2neg 3 neg | 4.3 | 60 | 100 | 2-3 | N/A |
| **8** | **Medtronic 3389** | **Left** | **Single monopolar** | **2neg** | **3.5** | **60** | **130** | N/A | N/A |
| **9** | **Medtronic 3389** | **Left** | **Double monopolar** | **1neg 2neg** | **2.4** | **60** | **80** | N/A | N/A |
| 10 | BSCI Cartesia | Left | Single monopolar | 5neg6neg7neg | 4.9 | 60 | 185 | 2 | NR |
| 11 | BSCI Cartesia | Left | Directional | 7neg | 3.4 | 60 | 185 | 1-2 | B |
| 12 | BSCI Cartesia | Left | Directional | 5neg | 2 | 60 | 179 | 1-2 | B |
| 13 | BSCI Cartesia | Left | Double monopolar | 2neg3neg4neg 5neg6neg7neg | 3.5 | 60 | 71 | 2-3 |  |
| 14 | BSCI Cartesia | Left | Directional | 6neg | 2.4 | 60 | 130 | 2 | B |
| 15 | BSCI Cartesia | Left | Directional | 2neg4neg | 4 | 60 | 185 | 3 | B-C |
| 16 | BSCI Cartesia | Left | Directional | 2neg5neg | 4.4 | 60 | 60 | 1-2 | C |
| 17 | BSCI Cartesia | Left | Single monopolar | 5neg 6neg 7neg | 0.6 | 60 | 130 | 2 |  |
| 18 | BSCI Cartesia | Left | Single monopolar | 5neg 6neg 7neg | 1.6 | 60 | 130 | 2 |  |
| 19 | BSCI Cartesia | Left | Directional | 7neg | 1.6 | 60 | 130 | 2-3 | C |
| 20 | BSCI Cartesia | Left | Directional | 6neg | 3.3 | 40 | 130 | 1-2 | B |
| 21 | BSCI Cartesia | Left | Directional | 7neg | 3.5 | 40 | 104 | 2 | C |
| 22 | BSCI Cartesia | Left | Directional | 5neg8neg | 5 | 60 | 185 | 1-2 | A |
| 23 | BSCI Cartesia | Left | Directional bipolar | 7neg 8pos | 5.7 | 60 | 185 | 1-2 | A-C |
| 24 | BSCI Cartesia | Left | Directional | 7neg8neg | 5 | 60 | 185 | 1-2 | A |
| 25 | BSCI Cartesia | Left | Directional | 5neg | 2.7 | 60 | 185 | 2-3 | C |
| 26 | BSCI Cartesia | Left | Directional | 6neg | 4.2 | 60 | 130 | 2-3 | B-C |
| 27 | Abbott directional | Left | Single monopolar | 3ABCneg | 1.6 | 60 | 130 | 1-2 |  |
| 28 | BSCI Cartesia | Left | Directional | 2neg | 3.2 | 60 | 130 | 1-2-3 | A |
| 29 | BSCI Cartesia | Left | Single monopolar | 5neg 6neg 7neg | 4 | 60 | 79 | 2-3 |  |
| 30 | BSCI Cartesia | Left | Single monopolar | 2neg3neg4neg | 3.7 | 60 | 130 | 2 |  |
| 31 | Abbott directional | Left | Double monopolar | 2AnegBnegCneg 3AnegBnegCneg | 6 | 60 | 60 | 2 |  |
| 32 | BSCI Cartesia | Left | Directional | 5neg | 2.9 | 60 | 185 | 1-2 | B-C |
| **33** | **Abbott directional** | **Left** | **Directional** | **3BnegCneg** | **2.2** | **40** | **120** | N/A | N/A |
| 34 | Medtronic 3389 | Left | Single monopolar | 2neg | 2.7 | 60 | 130 | 2-3 | N/A |
| 35 | BSCI Cartesia | Left | Double monopolar | 2AnegBnegCneg 3AnegBnegCneg | 2.8 | 60 | 130 | 1-2 |  |
| 1 | Medtronic 3389 | Right | Bipolar | 8neg 10pos | 3.9 | 60 | 150 | 3-4 | N/A |
| 2 | Medtronic 3389 | Right | Triple monopolar | 9neg 10neg 11neg | 5.5 | 60 | 150 | 4 | N/A |
| 3 | Medtronic 3389 | Right | Bipolar | 9neg 10pos | 3.8 | 60 | 180 | 3-4 | N/A |
| 4 | Medtronic 3389 | Right | Double monopolar | 9neg 10neg | 3.3 | 60 | 180 | 1-2 | N/A |
| 5 | Medtronic 3389 | Right | Bipolar | 9neg 10pos | 4.7 | 60 | 130 | 3-4 | N/A |
| 6 | Medtronic 3389 | Right | Double monopolar | 9neg 10neg | 3.9 | 60 | 60 | 3-4 | N/A |
| 7 | Medtronic 3389 | Right | Double monopolar | 9neg 10neg | 3.3 | 60 | 100 | 3-4 | N/A |
| 8 | Medtronic 3389 | Right | Single monopolar | 10neg | 2.7 | 60 | 130 | 3-4 | N/A |
| 9 | Medtronic 3389 | Right | Bipolar | 10neg 11pos | 1.6 | 60 | 80 | 3-4 | N/A |
| 10 | BSCI Cartesia | Right | Single monopolar | 2neg3neg4neg | 3 | 60 | 185 | 2-3 |  |
| 11 | BSCI Cartesia | Right | Single monopolar | 5neg6neg7neg | 1.5 | 60 | 185 | 1-2 |  |
| 12 | BSCI Cartesia | Right | Single monopolar | 2neg3neg4neg | 1.4 | 60 | 179 | 1-2-3 |  |
| 13 | BSCI Cartesia | Right | Single monopolar | 5neg6neg7neg | 2.3 | 60 | 71 | 1-2 |  |
| 14 | BSCI Cartesia | Right | Directional | 7neg | 1.4 | 60 | 130 | 2-3 | B-C |
| 15 | BSCI Cartesia | Right | Single monopolar | 5neg6neg7neg | 2.2 | 60 | 185 | 2-3 |  |
| 16 | BSCI Cartesia | Right | Directional | 2neg | 2 | 60 | 60 | 2-3 | A-B |
| 17 | BSCI Cartesia | Right | Single monopolar | 5neg 6neg 7neg | 2.3 | 60 | 130 | 2 |  |
| 18 | BSCI Cartesia | Right | Single monopolar | 2neg3neg4neg | 1.6 | 60 | 130 | 3 |  |
| 19 | BSCI Cartesia | Right | Single monopolar | 2neg3neg4neg | 0.8 | 60 | 130 | 3-4 |  |
| 20 | BSCI Cartesia | Right | Directional | 7neg | 3.5 | 40 | 130 | 1-2 | A-C |
| 21 | BSCI Cartesia | Right | Single monopolar | 5neg6neg7neg | 1.2 | 60 | 104 | 2 |  |
| 22 | BSCI Cartesia | Right | Single monopolar | 5neg6neg7neg | 1.9 | 60 | 185 | 1-2 |  |
| 23 | BSCI Cartesia | Right | Single monopolar | 5neg6neg7neg | 2.6 | 60 | 185 | 2-3 |  |
| 24 | BSCI Cartesia | Right | Single monopolar | 5neg6neg7neg | 2.5 | 60 | 185 | 2-3 |  |
| 25 | BSCI Cartesia | Right | Directional | 5neg7neg | 4 | 60 | 185 | 2-3 | A-B |
| 26 | BSCI Cartesia | Right | Single monopolar | 5neg6neg7neg | 4.2 | 60 | 130 | 2-3 |  |
| 27 | Abbott directional | Right | Single monopolar | 11AnegBnegCneg | 2.1 | 60 | 130 | 1-2 |  |
| 28 | BSCI Cartesia | Right | Single monopolar | 2neg3neg4neg | 3 | 60 | 130 | 2-3 |  |
| 29 | BSCI Cartesia | Right | Single monopolar | 2neg3neg4neg | 1.9 | 60 | 79 | 3-4 |  |
| 30 | BSCI Cartesia | Right | Single monopolar | 2neg3neg4neg | 3.2 | 60 | 130 | 2-3 |  |
| 31 | Abbott directional | Right | Single monopolar | 10AnegBnegCneg | 3.1 | 60 | 60 | 3-4 |  |
| 32 | BSCI Cartesia | Right | Directional | 6neg | 1.9 | 60 | 185 | 1-2 | B |
| 33 | Abbott directional | Right | Single monopolar | 11AnegBnegCneg | 0.3 | 60 | 130 | 1-2 |  |
| 34 | Medtronic 3389 | Right | Single monopolar | 10neg | 4 | 60 | 130 | 2-3 | N/A |
| 35 | BSCI Cartesia | Right | Directional | 11Cneg | 2.8 | 40 | 130 | 2-3 | B-C |

Supplementary Table 2: Comparison of demographic and clinical features at baseline between contact level IGP/CP-concordant vs IGP/CP-discordant patients. Mann-Whitney or chi-squared tests were used as appropriate. Abbreviations: UPDRS = Unified Parkinson's Disease Rating Scale; LEDD = Levodopa Equivalent Daily Dose.

| **Variable, mean (± SD)** | **contact level IGP/CP- concordant (N=25)** | | **contact level IGP/CP-discordant (N=7)** | ***p*** |
| --- | --- | --- | --- | --- |
| **Age at DBS implant** | 55.1 (± 8.2) | 58.3 (± 3.7) | | 0.3 |
| **Disease duration at DBS implant** | 11.2 (± 4.8) | 12.4 (± 7.5) | | 0.6 |
| **Male sex, n/N (%)** | 17/25 (68.0%) | 5/7 (71.4%) | | 0.9 |
| **Hoehn & Yahr** | 2.8 (± 0.7) | | 2.9 (± 0.2) | 0.6 |
| **UPDRS I total** | 1.3 (± 1.1) | | 2.6 (± 1.5) | 0.08 |
| **UPDRS II total** | 12.0 (± 5.7) | | 13.4 (± 5.3) | 0.6 |
| **UPDRS III** |  | |  |  |
| **Medication-ON** | 19.4 (± 7.9) | | 15.7 (± 2.3) | 0.2 |
| **Medication-OFF** | 30.3 (± 3.5) | | 42.7 (± 15.1) | **0.042** |
| **UPDRS III axial subscore** |  | |  |  |
| **Medication-ON** | 4.3 (± 2.6) | | 4.0 (± 1.4) | 0.8 |
| **Medication-OFF** | 9.5 (±5.6) | | 7.4 (±1.0) | 0.3 |
| **UPDRS IV total** | 7.3 (± 3.2) | | 7.8 (± 3.0) | 0.8 |
| **LEDD** | 1337.9 (± 495.1) | | 1223.1 (± 335.4) | 0.6 |

Supplementary Table 3: Results of separate multivariable linear regressions investigating motor outcomes in our cohort. Outcome variables of interest were Δ scores  ( OFF-medication/ON-stimulation follow up value – OFF-medication baseline value); in each single-entry model, IGP/CP concordance was entered as the main predictor, while gender and age at surgery were included as covariates; contact level concordance analysis was also adjusted for baseline OFF-medication UPDRS-III. Significant results are marked with an asterisk and highlighted in bold.

| **Outcome** | | **F (*p*)** | **R^2^ (Adj)** | **IGP/CP contact level concordance  (B [95% CI]; *p*)** | **Gender  (B [95% CI]; *p*)** | **Age at surgery  (B [95% CI]; *p*)** |
| --- | --- | --- | --- | --- | --- | --- |
| **Δ Hoehn & Yahr** | 1.8 (0.2) | | 0.2 (0.1) | 0.2 [–0.5; 0.9]; 0.5 | -0. 1 [–0.7; 0.5]; *0.7* | 0.04 [0.003; 0.08]; 0.033 |
| **Δ UPDRS I** | 1.0 (0.4) | | 0.3 (0.1) | 1.7 [–0.5; 3.8]; 0.1 | 0.4 [–1.4; 2.2]; *0.6* | 0.05 [-0.07; 0.2]; 0.4 |
| **Δ UPDRS II** | 0.7 (0.6) | | 0.1 (-0.0) | -0.7 [–7.9; 6.5]; 0.8 | -4.1 [–10.1; 1.9]; 0.2 | -0.03 [-0.4; 0.4]; 0.9 |
| **Δ UPDRS III** |  | |  |  |  |  |
| **total** | 0.8 (0.5) | | 0.1 (-0.0) | -8.9 [–22.2; 4.3]; 0.2 | 4.6 [–7.5; 16.6]; *0.4* | -0.1 [-0.9; 0.6]; 0.8 |
| **axial** | 0.4 (0.7) | | 0.0 (-0.1) | -1.0 [–5.6; 3.6]; 0.7 | 2.1 [–2.1; 6.3]; *0.3* | -0.03 [-0.3; 0.2]; 0.8 |
| **Δ UPDRS IV** | 0.8 (0.5) | | 0.1 (0.0) | 0.5 [–4.0; 5.0]; 0.8 | -2.0 [–5.6; 1.6]; 0.3 | -0.1 [-0.3; 0.1]; 0.3 |
| **Δ LEDD** | 0.9 (0.4) | | 0.1 (-0.0) | -291 [–739; 156], 0.2 | 46.8 [–297; 441], *0.8* | -14.7 [–39; 10], *0.2* |
| **Outcome** | **F (p)** | | **R2 (Adj)** | **IGP/CP directionality concordance  (B [95% CI]; p)** | **Gender  (B [95% CI]; p)** | **Age at surgery  (B [95% CI]; p)** |
| **Δ Hoehn & Yahr** | 1.3 (0.3) | | 0.2 (0.1) | 0.3 [–0.9; 1.6]; 0.6 | -0. 7 [–2.0; 0.6]; 0.3 | 0.04 [-0.04; 0.1]; 0.3 |
| **Δ UPDRS I** | 1.0 (0.4) | | 0.2 (0.0) | 2.2 [–0.6; 5.0]; 0.1 | -1.2 [–4.0; 1.7]; 0.4 | 0.03 [-0.2; 0.1]; 0.1 |
| **Δ UPDRS II** | 2.3 (0.1) | | 0.4 (0.2) | 6.2 [–2.1; 14.4]; 0.1 | -8.6 [–17.1; -0.2]; 0.046 | 0.1 [-0.4; 0.6]; 0.6 |
| **Δ UPDRS III** |  | |  |  |  |  |
| **total** | 4.2 (***0.036**) | | 0.6 (0.4) | -16.2 [–31.7; –0.8]; ***0.042** | 1.8 [–14.1; 17.7]; 0.8 | 1.3 [0.4; 2.3]; ***0.012** |
| **axial** | 4.4 (***0.033**) | | 0.6 (0.4) | -5.8 [–10.4; –1.1]; ***0.021** | 2.7 [–2.1; 7.5]; 0.2 | 0.4 [0.1; 0.7]; ***<0.01** |
| **Δ UPDRS IV** | 0.6 (0.9) | | 0.1 (-0.1) | 1.8 [–4.8; 8.4]; 0.6 | -3.7 [–10.4; 3.0]; 0.2 | -0.1 [-0.5; 0.3]; 0.5 |
| **Δ LEDD** | 0.2 (0.9) | | 0.0 (-0.2) | 15.8 [–681; 713], 1 | -145.8 [–850; 561], 0.7 | 9.0 [–34.6; 52.5], 0.7 |

Supplementary Table 4: Comparison of demographic and clinical features at baseline between directionality IGP/CP-concordant vs IGP/CP-discordant patients. Mann-Whitney or chi-squared tests were used as appropriate. Abbreviations: UPDRS = Unified Parkinson's Disease Rating Scale; LEDD = Levodopa Equivalent Daily Dose.

| **Variable, mean (± SD)** | **directionality IGP/CP- concordant (N=10)** | | **directionality IGP/CP-discordant (N=6)** | ***p*** |
| --- | --- | --- | --- | --- |
| **Age at DBS implant** | 55.6 (± 7.5) | 52.3 (± 5.8) | | 0.4 |
| **Disease duration at DBS implant** | 10.4 (± 4.6) | 9.3 (± 3.2) | | 0.6 |
| **Male sex, n/N (%)** | 5/10 (50.0%) | 0/6 (0%) | | **0.037** |
| **Hoehn & Yahr** | 2.9 (± 0.9) | | 2.5 (± 0.5) | 0.5 |
| **UPDRS I total** | 1.6 (± 1.3) | | 1.5 (± 1.4) | 0.8 |
| **UPDRS II total** | 12.7 (± 5.3) | | 12.6 (± 6.4) | 0.8 |
| **UPDRS III** |  | |  |  |
| **Medication-ON** | 19.6 (± 5.1) | | 14.2 (± 7.1) | 0.1 |
| **Medication-OFF** | 44.4 (± 15.5) | | 31.8 (± 10.3) | 0.4 |
| **UPDRS III axial subscore** |  | |  |  |
| **Medication-ON** | 3.6 (± 1.6) | | 2.8 (± 1.8) | 0.7 |
| **Medication-OFF** | 9.4 (±5.3) | | 5.8 (±2.8) | 0.2 |
| **UPDRS IV total** | 8.1 (± 4.2) | | 7.0 (± 2.4) | 0.6 |
| **LEDD** | 1180.0 (± 559.8) | | 1286.8 (± 248.9) | 0.7 |

**Supplementary Figure**

Supplementary Figure 1: Examples of assessments of level contact and directionality CP/IGP concordance from our cohort. Lead and contact position in the 3D anatomical reconstruction of the basal ganglia anatomy and estimation of stimulated tissue as displayed in Guide^TM^ XT software (Boston Scientific, Marlborough, MA). Green: subthalamic nucleus, red: red nucleus, blue: substantia nigra, yellow: stimulated tissue. (A) Concordance between CP-activated and IGP-suggested stimulation contact level; (B) Discordant CP-activated and IGP-suggested stimulation contact level; (C) Concordance between CP-activated and IGP-suggested stimulation directionality; (D) Discordant CP-activated and IGP-suggested stimulation directionality; (E) both middle contacts reside inside the STN, therefore are considered IGP-suggested; similarly, since A (2/5) and C (4/7) directional segments point towards the motor portion of the STN they are both considered IGP-suggested. Figure modified from Rigon et al., Journal of Parkinson's disease vol. 15,2 (2025): 409-420. doi:10.1177/1877718X241305725

**Supplementary Methods**

**Surgery**

Leads were placed using the NexFrame system (Medtronic, Inc., Minneapolis, MN). Targeting was performed using the StealthStation S8 (Medtronic). Electrodes placement was monitored intraoperatively with microelectrode recordings and O-arm 3D fluoroscopic imaging system (Medtronic). The number of tracks performed and track placement depended on the electrophysiological findings from the previous track. Unit recordings were performed with a Leadpoint 4 system (Medtronic). Intraoperative microstimulations were used to assess the therapeutic as well as side effects thresholds. After targeting the correct location, DBS leads were implanted. All implanted leads had four electrode levels, either with all four full-ring shaped contacts (Medtronic 3389) or with two annular contacts at the proximal and distal level and two central electrode levels with three segmented contacts each (Boston Scientific Cartesia DB2202; Abbott/St Jude directed 6172). The implantable pulse generator placement was performed under general anesthesia after lead placement.
